# High Cell Density Upregulates Calcium Oscillation by Increasing Calcium Store Content via Basal Mitogen-Activated Protein Kinase Activity

**DOI:** 10.1371/journal.pone.0137610

**Published:** 2015-09-23

**Authors:** Mitsuhiro Morita, Akira Nakane, Yuki Fujii, Shohei Maekawa, Yoshihisa Kudo

**Affiliations:** 1 Department of Biology, Kobe University Graduate School of Science, Kobe, Japan; 2 Laboratory of Cellular Neurobiology, School of Life Science, Tokyo University of Pharmacy and Life Science, Tokyo, Japan; Kinki University School of Pharmaceutical Sciences, JAPAN

## Abstract

Calcium releases of non-excitable cells are generally a combination of oscillatory and non-oscillatory patterns, and factors affecting the calcium dynamics are still to be determined. Here we report the influence of cell density on calcium increase patterns of clonal cell lines. The majority of HeLa cells seeded at 1.5 x 10^4^/cm^2^ showed calcium oscillations in response to histamine and ATP, whereas cells seeded at 0.5 x 10^4^/cm^2^ largely showed transient and sustained calcium increases. Cell density also affected the response of HEK293 cells to ATP in a similar manner. High cell density increased the basal activity of the mitogen-activated protein (MAP) kinase and calcium store content, and both calcium oscillation and calcium store content were down-regulated by a MAP kinase inhibitor, U0126. Thus, MAP kinase-mediated regulation of calcium store likely underlie the effect of cell density on calcium oscillation. Calcium increase patterns of HeLa cells were conserved at any histamine concentrations tested, whereas the overexpression of histamine H1 receptor, which robustly increased histamine-induced inositol phospholipid hydrolysis, converted calcium oscillations to sustained calcium increases only at high histamine concentrations. Thus, the consequence of modulating inositol phospholipid metabolism was distinct from that of changing cell density, suggesting the effect of cell density is not attributed to inositol phospholipid metabolism. Collectively, our results propose that calcium increase patterns of non-excitable cells reflect calcium store, which is regulated by the basal MAP kinase activity under the influence of cell density.

## Introduction

A wide variety of neurotransmitters, hormones and bioactive lipid metabolites has been shown to induce oscillatory intracellular calcium mobilization in non-excitable cells [1]. The majority of these molecules elicit inositol 1,4,5-trisphosphate (IP_3_) production and subsequent calcium releases from IP_3_ receptors on intracellular calcium store [2, 3]. This mechanism, known as IP_3_-induced calcium release, can have various patterns, including transient, sustained and oscillatory [4]. Calcium oscillations have been reported to enhance calcium dependent cellular processes, including secretion [5], enzyme activation [6] and gene expression [7]. Thus, calcium oscillation has been recognized as a fundamental issue for understanding diverse cellular functions, and extensively studied using both experimental and theoretical approaches [1, 8, 9]. Preceding studies have suggested the calcium dependences of IP_3_ receptors [10, 11] or IP_3_ metabolizing enzymes [12, 13] as components of a complex mechanism generating calcium oscillation, whereas cellular IP_3_ and calcium concentrations may show correlated oscillation patterns [14].

Even though a number of models have been proposed, the mechanisms underlying calcium oscillation is still an issue of controversial discussions [8, 15, 16]. One of the problems retarding the progress of this research is the heterogeneity of calcium increase patterns of cell lines. Even the histamine-induced calcium increases in HeLa cells, one of the most widely used clonal cell lines, were the mixture of heterogeneous calcium increase patterns [17, 18]. This heterogeneity has caused the difficulties in molecular biological approaches and of data comparison between different research groups. Without understanding the causality for the heterogeneity, the experimental approaches to calcium oscillation are limited by the insufficient reliability.

In the present study, we hypothesized that the pattern of calcium increase in cell lines, including HeLa cells, is affected by the cell culture environment, and screened for culture conditions in which HeLa cells preferentially showed calcium oscillation. As results, we have found cell density is the key environmental factor affecting calcium increase patterns. Moreover, our further analyses have demonstrated that the effect of cell density is attributed to the modulation of calcium store, rather than inositol phospholipid metabolism, via mitogen-activated protein (MAP) kinase activity.

## Materials and Methods

### Recombinant DNA

Expression vectors containing fusion proteins of the cyan and yellow variants of enhanced green fluorescent protein (EGFP) and the pleckstrin homology domain (PHD) derived from rat PLCδ1 were constructed and designated pCFP-PHD and pYFP-PHD, as described previously [19]. Histamine H1 receptor cDNA [20] was obtained by PCR amplification from bovine cDNA (Quick-Clone, BD bioscience, San Jose, CA) and used to construct an expression vector, pME-H1 using the SRα promoter [21]. An expression vector for EGFP, pEGFP-C1, was purchased from BD Bioscience.

### Cell culture and transfection

HeLa cells (ATCC) were seeded, at the densities indicated, on 12-mm diameter cover slips in Minimum Essential Medium Alpha Medium (Invitrogen, Gaithersburg, MD) containing 10% fetal calf serum (FCS, Equitech-Bio, Ingram, TX). Cells were transfected with plasmids using Lipofectin (Invitrogen) and cultured for 48–72 h to allow expression of exogenous cDNA. To identify HeLa cells expressing exogenous H1 receptor by calcium imaging, pME-H1 was co-transfected with pEGFP-C1. For FRET imaging pCFP-PHD and pYFP-PHD were co-transfected, with or without pME-H1. HEK293 cells (ATCC) were seeded in Dulbecco’s Modified Eagle’s Medium (DMEM Asahi Technoglass, Funabashi, Japan) containing 10% fetal calf serum (FCS).

### Imaging

Extracellular basal salt solution (BSS; 130 mM NaCl, 5.4 mM KCl, 5.5 mM glucose, 2 mM CaCl_2_, 1 mM MgCl_2_, 20 mM HEPES, pH 7.4) was used for all physiological experiments. For calcium imaging, cells were incubated for 45 min at 30°C in BSS containing Fura2-AM (7.5 μM; Dojin-kagaku, Kumamoto, Japan), washed three times, and incubated at room temperature for 20 min before imaging. During each step, sulfinpyrazone (100 μM) was added to BSS after the repeated washing. Fluorescence images were obtained using an IX70 inverted microscope, fitted with an OSP-EXA filter exchanger (Olympus, Tokyo, Japan) and a C6790 CCD camera (Hamamatsu Photonics, Hamamatsu, Japan), and analyzed using AQUACOSMOS software (Hamamatsu Photonics). For FRET imaging, the fluorescence was split by a W-View dichroic mirror system (Hamamatsu Photonics) equipped with a dichroic mirror (510LP) and 480DF30 and 535DF25 barrier filters for YFP-PHD and CFP-PHD, respectively. Calcium and inositol phospholipid hydrolysis were expressed as changes in the ratios of fluorescence intensities (ΔR). For calcium imaging, the fluorescence ratio was calculated by dividing the fluorescence intensity at 510 nm following excitation at 340 nm by that at 380 nm. For inositol phospholipid hydrolysis imaging, the fluorescence ratio was calculated by dividing the fluorescence intensity of YFP-PHD (535 nm) by that of CFP-PHD (489 nm), both of which were excited at 430 nm.

### Western blotting

Extracts of HeLa cells were prepared by adding 200 μl of SDS-PAGE sample buffer (50 mM Tris HCl, pH 6.8, 2% SDS, 10% glycerol, 0.1 M dithiothreitol, 1% bromophenol blue) to cells in each well of a 6-well plate, scraping off the cells, and sonicating the suspension. A 10 μl aliquot of extract was loaded onto each well of an SDS polyacrylamide gel, which was electrophoresed and transferred to a PVDF filter (Amersham, Buckinghamshire, UK), and each filter was incubated with mouse monoclonal antibody against phosphorylated extracellular signal-regulated kinase, ERK (2 μg/ml, Sigma) or rabbit polyclonal anti-ERK antibody (0.6 μg/ml, Sigma). Horseradish peroxidase-conjugated anti-mouse IgG (Amersham) and ECL Plus (Amersham) were used to detect all bound primary antibodies. Chemiluminescence was captured as digital images, using a CCD camera, AE-6972FC (Atto, Tokyo, Japan), and total intensities of the two bands corresponding to ERK1 and ERK2, were quantified using Photoshop (Adobe systems, San Jose, CA).

### Materials

All chemicals were from Sigma (St. Louis, MO), unless indicated otherwise. A mouse monoclonal anti-bFGF antibody, bFM-1 was purchased from Upstate, Lake Placid, NY, USA.

### Data analysis and statistics

Results from statistical analysis were presented as Means ± S.E.M. obtained from more than three experiments. Experimental groups were compared by Student's t-test or one-way analysis of variance (ANOVA) with Newman—Keuls post-hoc test, as appropriate.

## Results

### Effects of cell density

**Fig 1:**
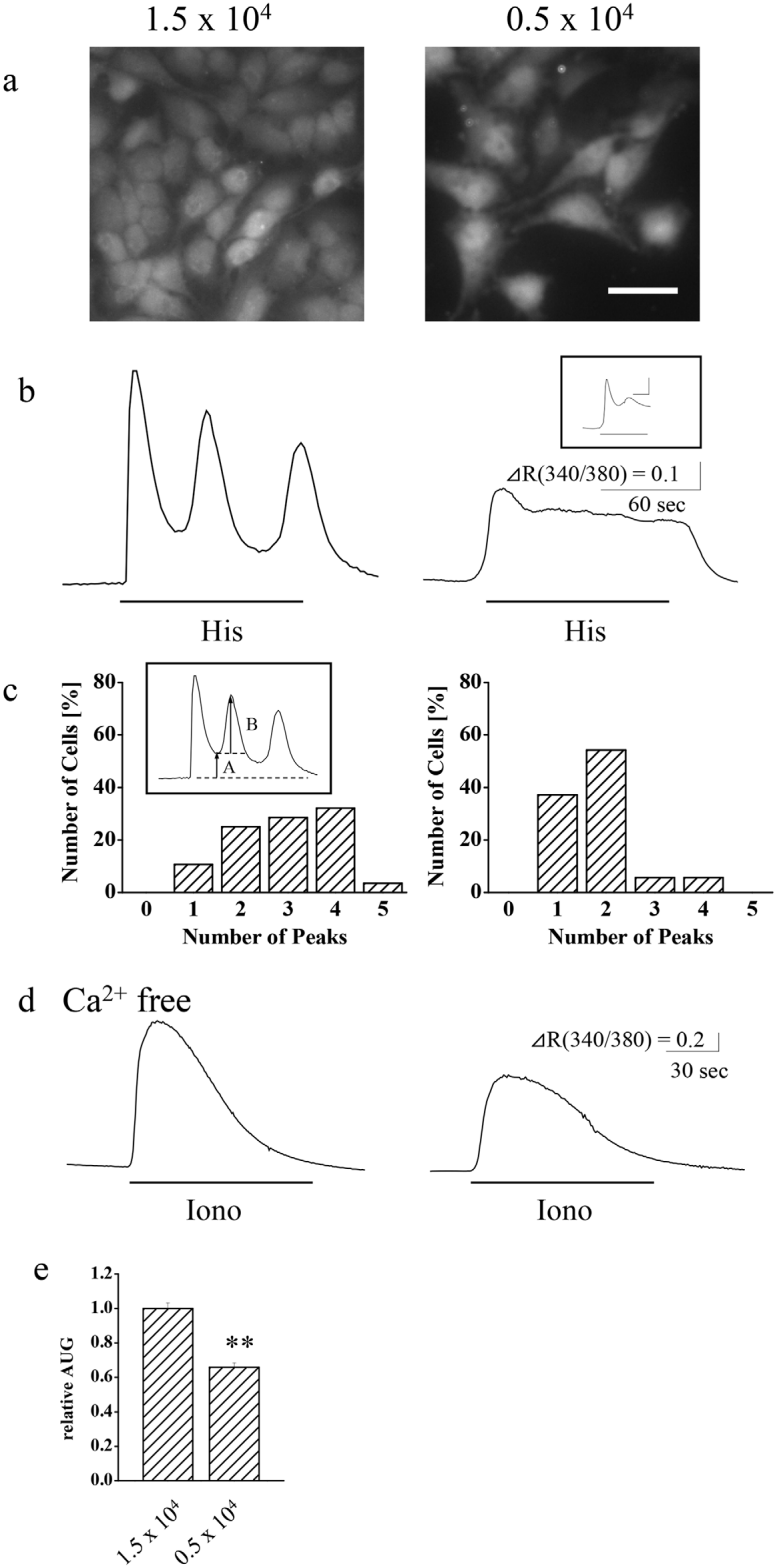
Effects of cell density on calcium release patterns in HeLa cells. Cells were seeded at a density of 1.5 x 10^4^ or 0.5 x 10^4^ cells/cm^2^. (a) Fluorescent images of Fura2-loaded HeLa cells excited at 380 nm. Bar, 25 μm. (b) Representative patterns and (c) peak histograms of calcium increase in response to stimulation with 30 μM histamine (His; 2 min, under lines) are shown. The scale bars in (a) inset indicate 30 sec and ΔR(340/380) = 0.5. (d) Representative patterns and (e) comparison of calcium store content from the area under the graph (AUG) of calcium release in response to treatment with 2.5 μM ionomycin (Iono; under lines) in the absence of extracellular calcium. Extracellular solution was changed to calcium free BSS 30 second prior to ionomycin stimulation. ** p < 0.01, n = 14–36 cells.

HeLa cells were seeded at different cell densities and cultured for 48–96 h, and their calcium increase patterns in response to 30 μM histamine were assayed. Cells seeded at 1.5 x 10^4^/cm^2^ showed an oscillatory calcium response, whereas those seeded at 0.5 x 10^4^/cm^2^ showed a transient and sustained pattern (Fig 1a, 1b and 1c). In the present study, peaks of calcium oscillations were defined as maximum of Fura2 fluorescent ratio (340/380) change, whose amplitude (B in Fig 1c inset) is more than 20% of the preceding minimum value (A in Fig 1c inset). Of the cells seeded at 0.5 x 10^4^/cm^2^, 54% showed a second peak, primarily as a small peak at the beginning of the sustained phase (Fig 1b, inset). On average, during 2 min of stimulation, 2.9 ± 0.2 peaks (n = 28 cells) were observed for cells seeded at 1.5 x 10^4^/cm^2^, whereas significantly smaller number of peaks, 1.8 ± 0.1 (n = 36 cells, p < 0.01) were observed for cells seeded at 0.5 x 10^4^/cm^2^, indicating that HeLa cells seeded at high density showed stronger oscillatory tendency than those seeded at lower density, in response to histamine.

In order to test the possibility that cell density affects calcium increase pattern by modulating calcium store, we compared calcium store contents by measuring ionomycin-induced calcium releases in the absence of extracellular calcium, as described previously [15] (Fig 1c and 1d). As a result, the calcium increase significantly decreased to 66 ± 3%, when the seeding cell density was reduced from 1.5 x 10^4^/cm^2^ (n = 14 cells) to 0.5 x 10^4^/cm^2^ (n = 14 cells), indicating that calcium stores in HeLa cells were decreased when the cells were seeded at the lower density.

**Fig 2:**
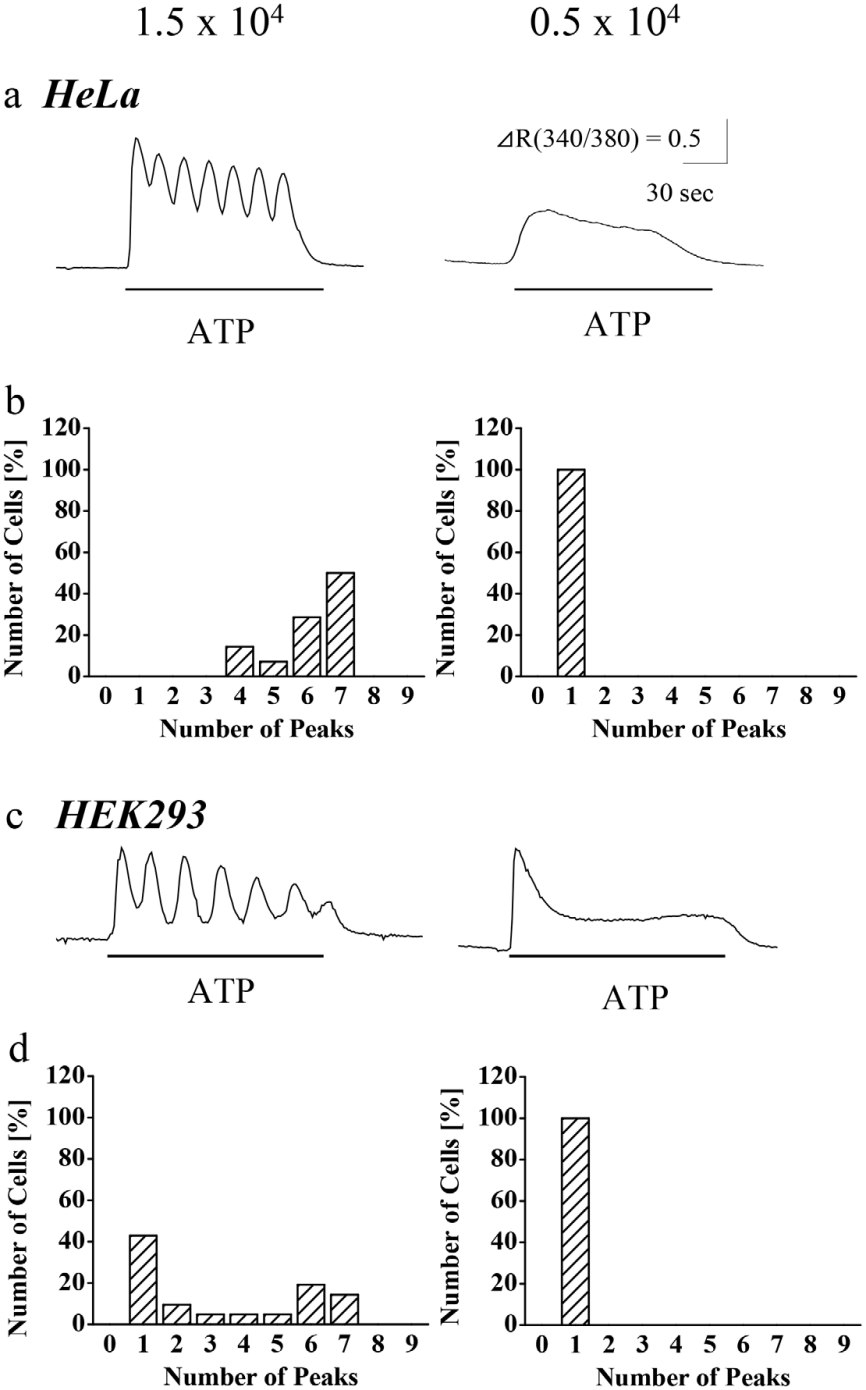
Effects of cell density to the calcium response patterns in HeLa cells (a, b) and HEK293 cells (c, d) in response to ATP. Cells were seeded at a density of 1.5 x 10^4^ or 0.5 x 10^4^ cells/cm^2^. Representative patterns (a and c) and peak histograms (b and d) of calcium increase in response to 100 μM ATP stimulation (2 min, under lines) are shown. n = 14–28 cells

The cell density-dependent change of calcium store contents likely affects calcium increase pattern to other stimuli in HeLa cell, and cell density may affect calcium increase pattern of other cell types. To test these possibilities, ATP-induced calcium increases in HeLa and HEK293 cells were assayed (Fig 2). As results, both cell lines responded to 100 μM ATP, with a response pattern similarly affected by cell density. When seeded at 0.5 x 10^4^ cells/cm^2^, both HeLa and HEK293 cells (n = 28 cells each) showed transient and sustained calcium increases. In contrast, when seeded at 1.5 x 10^4^ cells/cm^2^, both HeLa and HEK293 cell lines showed calcium oscillations, with 6.1 ± 0.2 (n = 28 cells) and 3.2 ± 0.5 (n = 28 cells) peaks, respectively. Thus, cell density is most likely a factor affecting calcium increase pattern of non-excitable cell in general.

### Involvement of MAP kinase cascade

**Fig 3:**
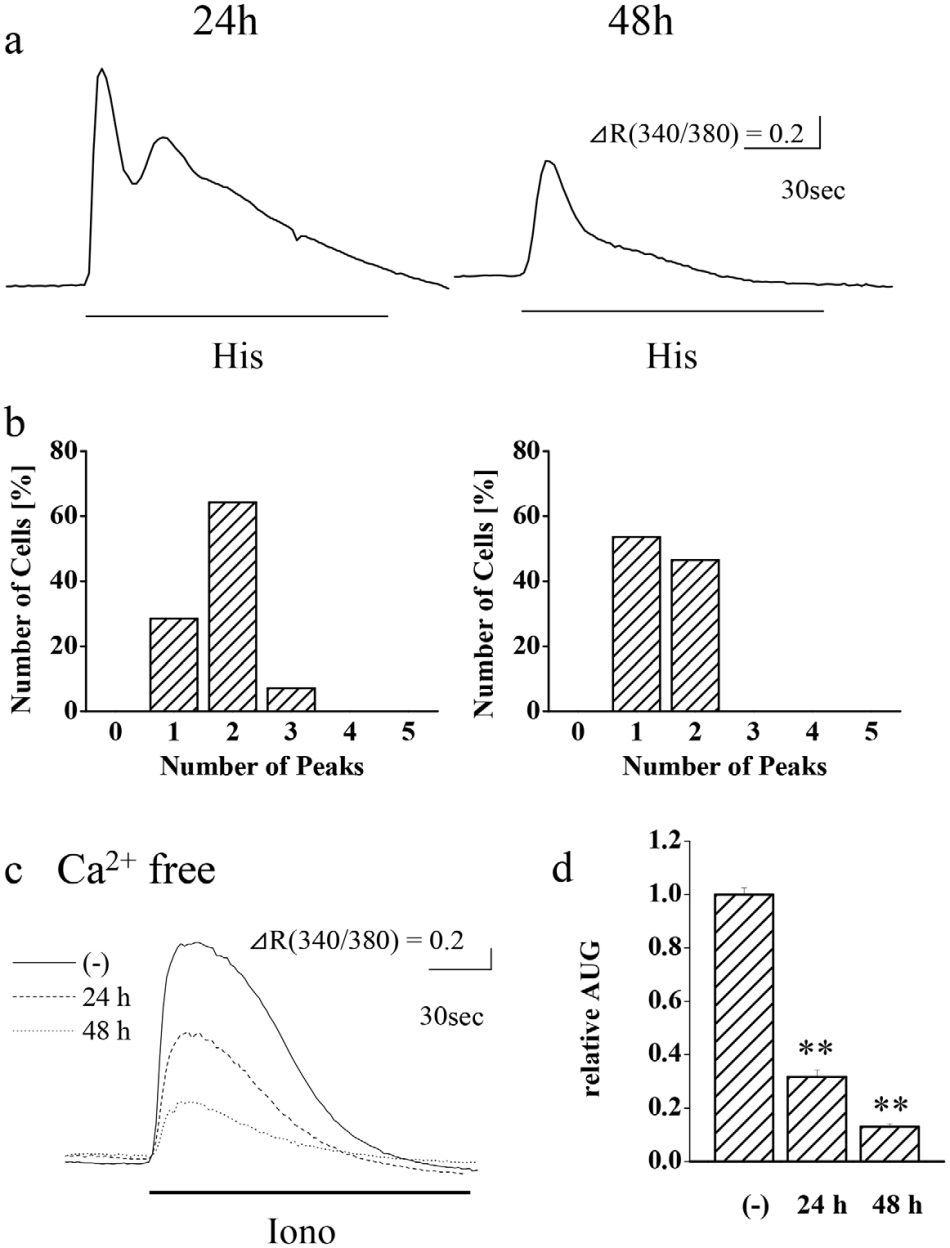
Involvement of MAP kinase cascade in the calcium increase pattern in HeLa cells. Cells were treated with 1 μM U0126 for 24 h or 48 h. (a) Representative patterns and (b) peak histograms of calcium increase in response to stimulation with 30 μM histamine (His; 2 min, under lines) are shown. (c) Representative patterns and (d) comparison of calcium store contents from the area under the graph (AUG) of calcium release in response to treatment with 2.5 μM ionomycin (Iono; under lines) in the absence of extracellular calcium. Extracellular solution was changed to calcium free BSS 30 sec prior to ionomycin stimulation. ** p < 0.01, n = 14–28 cells.

In order to test the possibility that the effect of cell density is attributed to growth signals, we examined whether MAP kinase cascade was involved in the conversion of calcium increase patterns. HeLa cells were seeded at 1.5 x 10^4^/cm^2^ and cultured for 24 h, after which the cells were cultured in the presence of a MEK inhibitor U0126 for 24 h or 48 h and then agonist-induced calcium changes were assayed (Fig 3). As results, the U0126 treatments for 24 h and 48 h significantly reduced the number of histamine-induced calcium peaks to 1.8 ± 0.1 (n = 28 cells) and 1.5 ± 0.1 (n = 28 cells), respectively, comparing with that of the non-treated cells in Fig 1 (2.9 ± 0.2 peaks, p < 0.01). The effect of U0126 was also examined on calcium store content, and the incubations with this inhibitor for 24 h and 48 h were found to significantly reduce the ionomycin-induced calcium release from 100 ± 2% (n = 28 cells) to 32 ± 3% (n = 28 cells) and 13 ± 1% (n = 28 cells), respectively. Since an acute U0126 treatment did not affect the calcium oscillation as shown later, it is suggested that U0126 does not alter the calcium release pattern by inhibiting histamine- or ionomycin-induced MEK activation, but by affecting either gene expression or the phosphorylation states of MAP kinase targets. Our findings indicate that U0126 reduces calcium store content and suppresses histamine-induced calcium oscillation in HeLa cells. Since the effects of U0126 were almost identical to those obtained by reducing cell density (Fig 1), our findings suggest that cell density affects the calcium increase pattern via the MAP kinase cascade.

**Fig 4:**
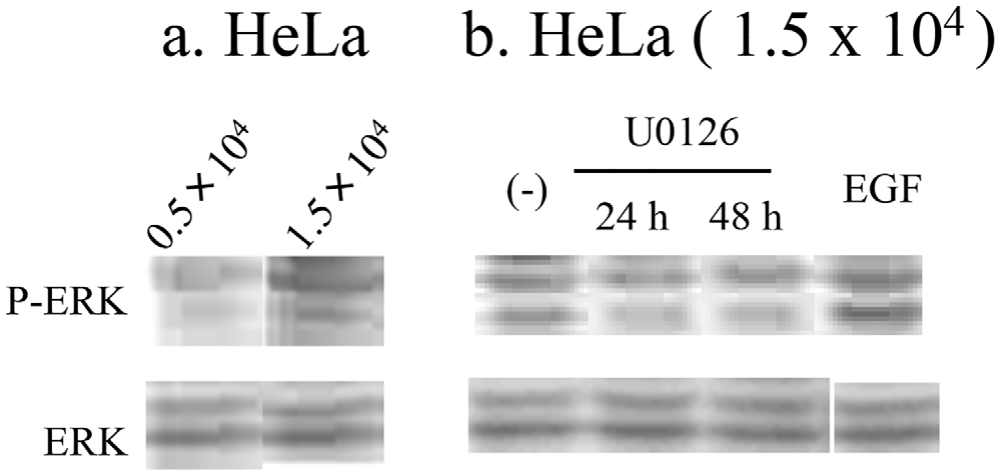
Basal phosphorylation level of ERK. Cell extracts were subjected to western blotting using anti-phosphorylated ERK (P-ERK) and anti-ERK (ERK) antibodies. (a) Basal phosphorylation level of ERK in HeLa cells seeded at 1.5 x 10^4^ and 0.5 x 10^4^ cells/cm^2^. (b) Effects of U0126 treatment (1 μM, 24 h or 48 h) and EGF stimulation (10 ng/ml, 5min) in HeLa cells seeded at 1.5 x 10^4^.

To confirm that MAP kinase activity is affected by seeding density, we measured ERK phosphorylation by Western blotting using anti-ERK and anti-phosphorylated ERK antibodies [22] (Fig 4). As results, the ratio of phosphorylated to total ERK of HeLa cells seeded at 0.5 x 10^4^/cm^2^ was significantly smaller (66.7 ± 13.3%, n = 3, p < 0.05) than those seeded at 1.5 x 10^4^/cm^2^ by quantifying the band intensities desitometrically (Fig 4a). The U0126 treatment, which suppressed the calcium oscillation of HeLa cells was examined for effects on the intrinsic phosphorylation levels of ERK. As results, 24 h and 48 h U0126 treatments reduced the ratio of phosphorylated to total ERK of HeLa cells seeded at 1.5 x 10^4^ cells/cm^2^ to 75.2% and 77.3% of the non-treated cells, respectively (Fig 4b left three lanes). As a control, HeLa cells seeded at 1.5 x 10^4^ cells/cm^2^ were treated with EGF, and the ratio of phosphorylated to total ERK was found to increase to 116.1% of non-treated cells (Fig 4b right). These results show ERK phosphorylation levels in HeLa cells are affected by seeding density, and the increased intrinsic ERK phosphorylation level of HeLa cells seeded at 1.5 x 10^4^ cells/cm^2^, which is below the level induced by EGF, is sensitive to the U0126 treatment. Therefore the intrinsic ERK phsophorylation levels in HeLa cells well correlate to calcium oscillations.

### Cell growth and calcium oscillation

**Fig 5:**
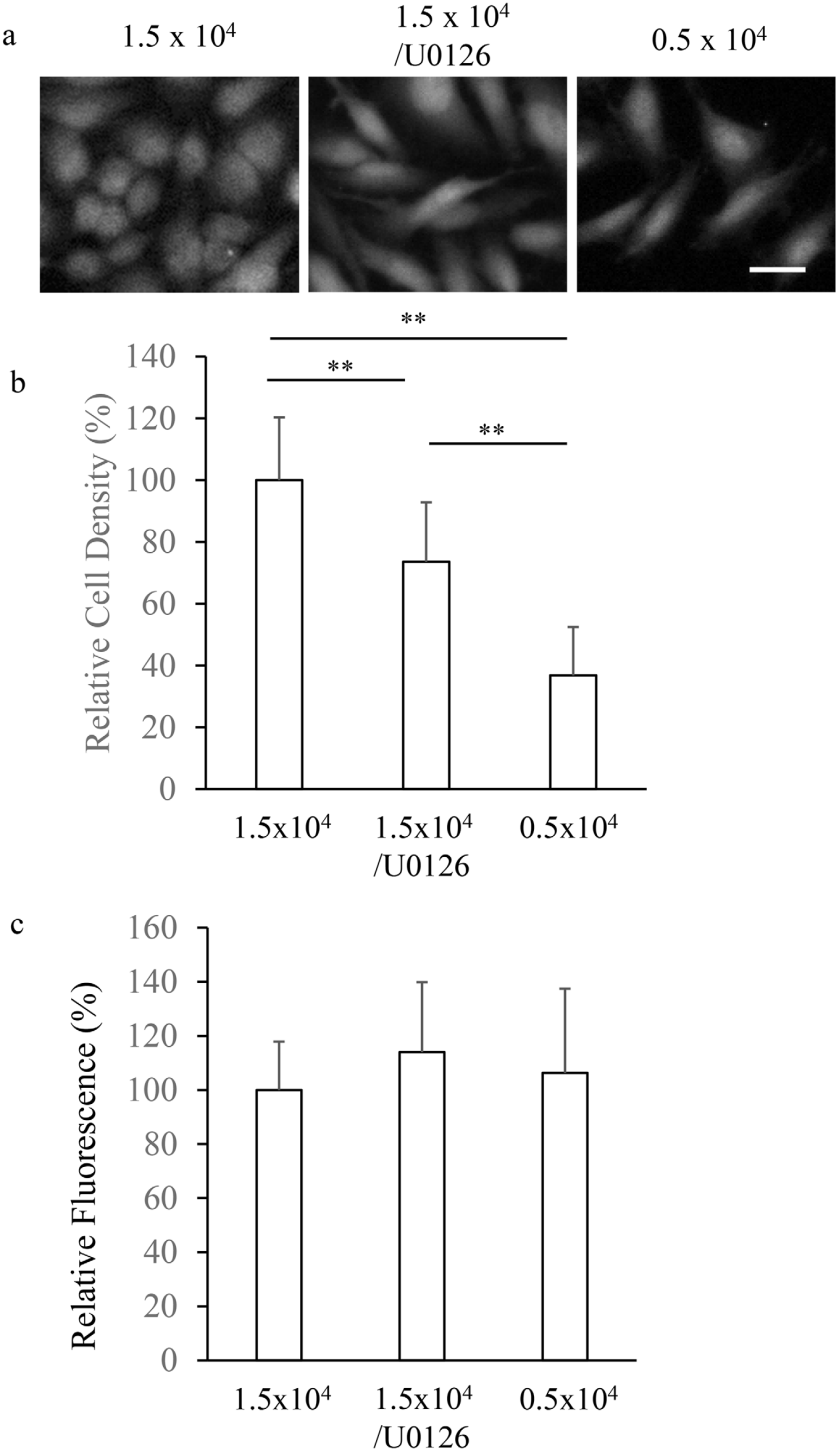
Effects of the MAP kinase inhibitor to cell density and Fura2 loading. HeLa cells were seeded at 1.5 x 10^4^ cells/cm^2^ or 0.5 x 10^4^ cells/cm^2^, and cultured in the presence or absence of 1 μM U0126 for 48 h. After loading Fura2-AM, fluorescent images were obtained by exciting at the isosbestic wave length of Fura2, 360 nm. (a) Representative fluorescent images. Bar, 25 μm. (b) Relative cell density obtained by counting Fura2-loaded cells in 433 x 330 μm imaging area. ** p < 0.01 (n = 3 images). (c) Relative Fura2 fluorescence. n = 20 cells

Since MAP kinase is involved in cell growth, it is assumed that U0126 alters growth states, which may affect the enzymatic activity for metabolizing acetoxymethyl ester, including Fura2-AM. If this is the case, U0126 affects calcium imaging results by altering the buffering capacity of calcium indicator due to the different efficiency of loading between growth states. In order to address this point, we examined the influence of seeding cell density and U0126 on the cell density and the Fura2 fluorescence at isobestic point of the HeLa cells 48 h after seeding. As shown in Fig 5a, HeLa cells seeded at 1.5 x 10^4^ cells/cm^2^ showed round morphology, whereas the morphology of cells seeded at 1.5 x 10^4^ cells/cm^2^ and treated with U0126, or cells seeded at 0.5 x 10^4^ cells/cm^2^ were fibrous. The density of cells seeded at 1.5 x 10^4^ cells/cm^2^ and grown for 48 h was significantly reduced to 73.6% by U0126 treatment, and the density of cells seeded at 0.5 x 10^4^ cells/cm^2^ and grown for the same period was further smaller down to 36.8% (Fig 5b). Since cell integrity was not affected either by seeding density or U0126 treatment so far as accessed by propidium iodide staining (data not shown), U0126 and low cell density less likely reduced cell density by cell death. Since HeLa cells seeded at 0.5 x 10^4^ cells/cm^2^ or treated with U0126 showed similar fibrous morphology, the down-regulation of calcium oscillation and calcium store content of these cells are likely reflect a state of HeLa cells of low growth rate due to a low basal MAP kinase activity. As shown in Fig 5c, the calcium independent Fura2 fluorescence was not significantly affected either by seeding cell density or U0126 treatment, suggesting that these culture conditions negligibly affected intracellular Fura2 concentration. Thus, the alteration of calcium oscillation by culture conditions less likely reflects the difference of calcium buffering capacity of calcium indicator.

### Pharmacological characterization of calcium oscillation

**Fig 6:**
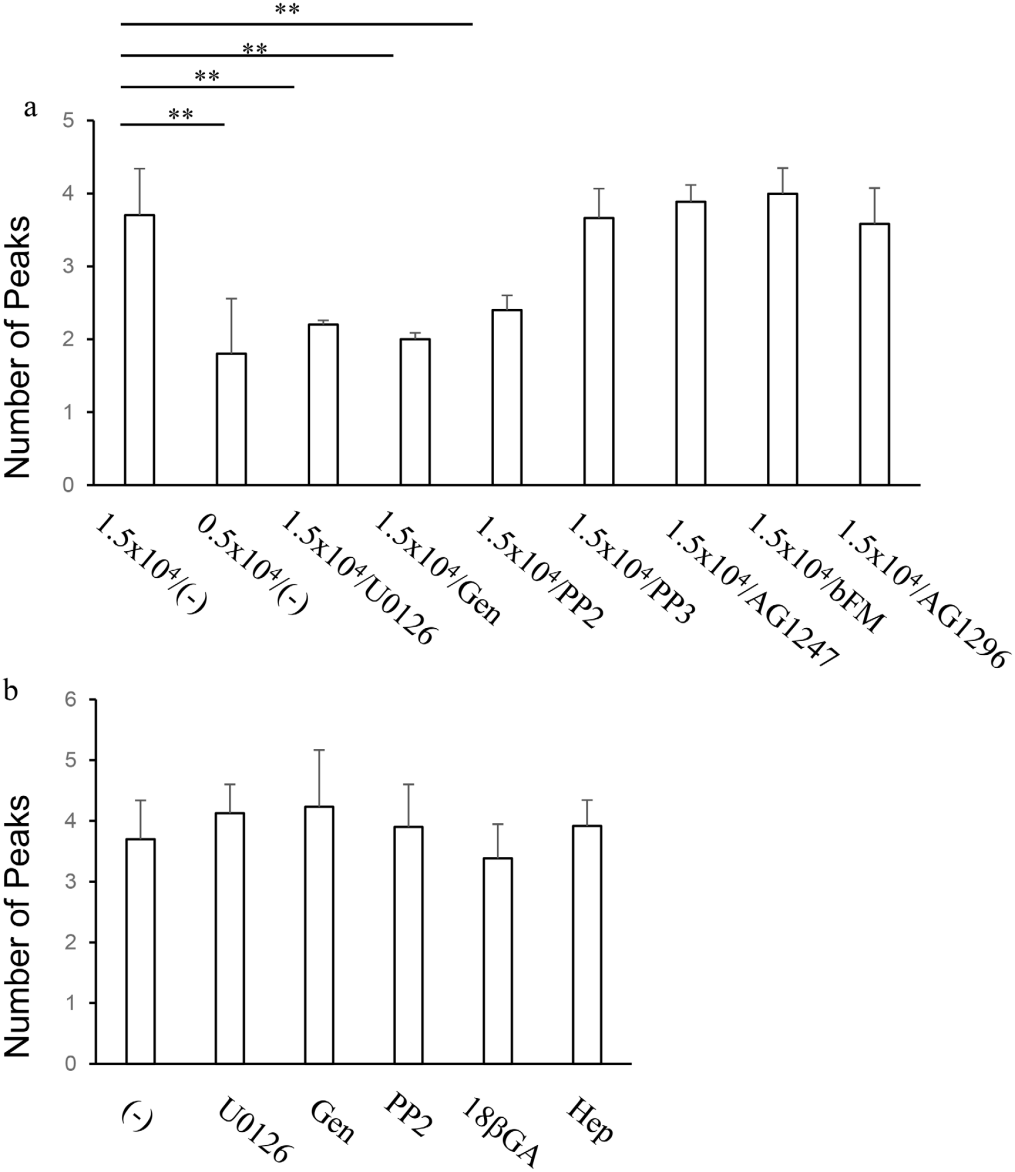
Pharmacological characterization of the calcium increase patterns of HeLa cells in response to histamine. (a) Effects of long-term pharmacological treatments during cell culture. Cells were seeded at 1.5 x 10^4^ cells/cm^2^ or 0.5 x 10^4^ cells/cm^2^, and cultured in the presence or absence or pharmacological agents, including 1 μM U0126, 30 μM Genistein (Gen), 10 μM PP2, 10 μM PP3, 10 μM AG1476, 10 μM AG1247 and 5 μg/ml anti-bFGF neutralizing antibody (bFM) for 48 h prior to calcium imaging experiments. Numbers of peaks of calcium increases during 2 min stimulation with 30 μM histamine were plotted. ** p < 0.01, n = 20 cells. (b) Effects of acute pharmacological treatments during calcium imaging. Cells were seeded at 1.5 x 10^4^ cells/cm^2^, cultured in normal medium for 48 h and subjected to calcium imaging. Each pharmacological treatment, including, U0126, Genistein, 10 μM 18 β-glycyrrhetinic acid (18βGA) and 2 mM heptanol (Hep) started 1 min prior to histamine stimulation. Numbers of peaks of calcium increases during 2 min stimulation were plotted. ** p < 0.01, n = 20 cells

The effect of cell density to the calcium oscillation was further examined pharmacologically. In order to characterize growth signaling pathways involved in the up-regulation of calcium oscillation, we have tested a tyrosine kinase inhibitor, Genistein, a selective inhibitor of Src-family tyrosine kinases, PP2 and its inactive analogue, PP3, an EGF receptor inhibitor, AG1467, a platelet-derived growth factor (PDGF) inhibitor, AG1296 and a neutralizing antibody against bFGF, bFM-1 by treating cells for 48 h prior to calcium imaging experiments. As shown in Fig 6a, Genistein and PP2 reduced the number of peaks of during calcium oscillation to the same extent as the low seeding density and U0126, whereas AG1467, AG1247 and bFM-2 did not affect the calcium oscillation. Thus, it has been suggested that Src-family tyrosine kinases are in the upstream of MAP kinase, and are involved in the up-regulation of calcium oscillation. And the possibility that this signaling cascade is initiated by autocrinal EGF-related growth factors, PDGF or bFGF has been excluded.

Even though cells had been extensively washed before calcium imaging, residual U0126, PP2 and Genistein in the cells might have directly affected calcium increase patterns. In order to test this possibility, inhibitor treatments started 1 min prior to histamine stimulation in calcium imaging experiments using HeLa cells cultured without inhibitors. As shown in Fig 6b, these inhibitors did not show direct influence on the number of peaks during calcium increases of HeLa cells seeded at 1.5 x 10^4^ cells/cm^2^, indicating that the direct effects of these inhibitors are negligible. We also examined the direct effects of gap junction blockers, because high cell density increase the contact between cells and up-regulates the gap junction mediated-interaction. As shown in Fig 6b, 18 β-glycyrrhetinic acid or heptanol did not affect the number of peaks, indicating that gap junction is less likely involved in the calcium oscillation HeLa cells seeded at 1.5 x 10^4^ cells/cm^2^.

### Inositol phospholipid hydrolysis during calcium oscillation

**Fig 7:**
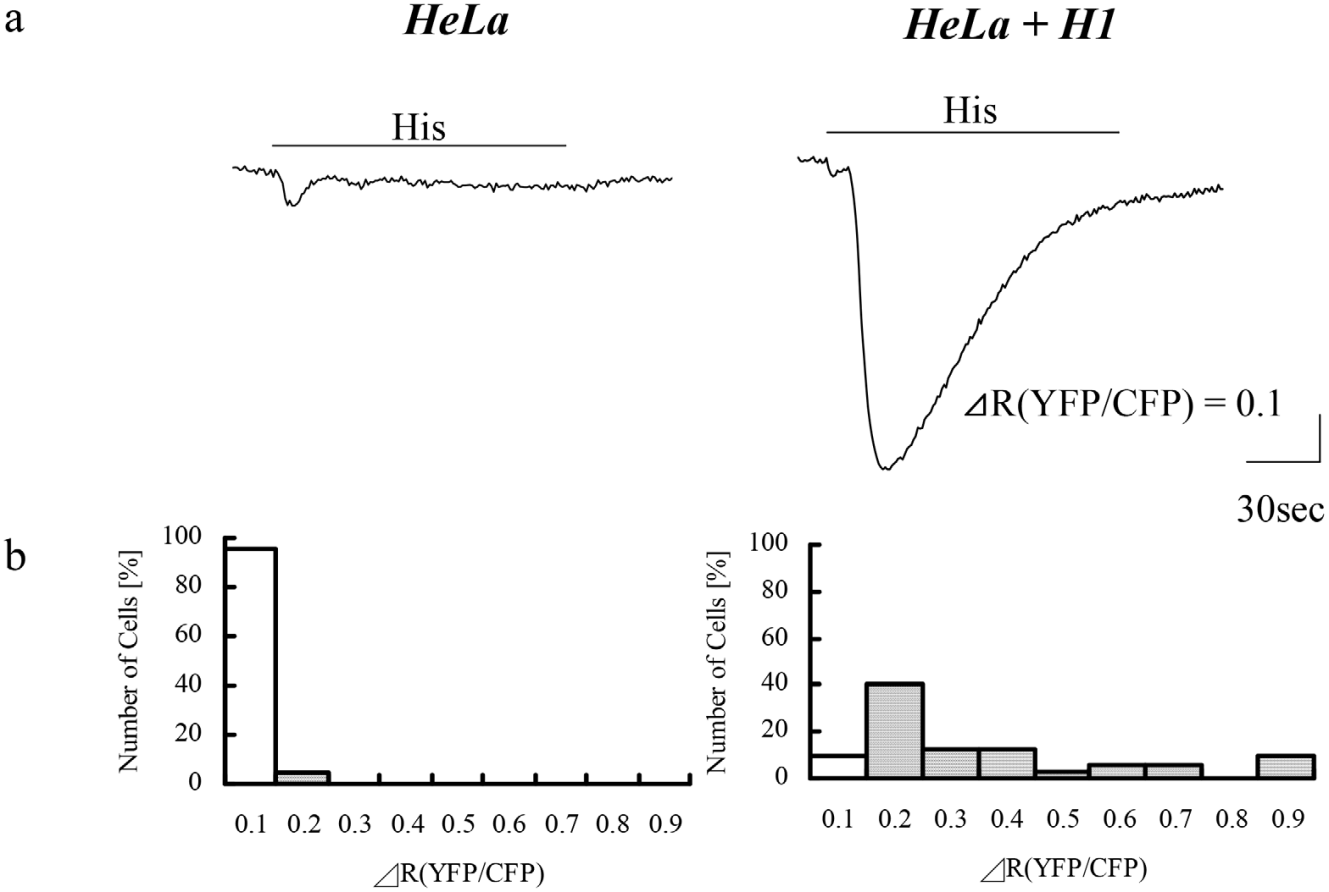
Histamine-induced inositol phospholipid hydrolysis in HeLa cells with or without exogenous histamine H1 receptor. HeLa were seeded at 1.5 x 10^4^, and subjected to FRET-based fluorescence imaging of inositol phospholipid hydrolysis. Native HeLa cells (HeLa) or HeLa cells expressing exogenous H1 receptor (HeLa + H1) were stimulated with 30 μM histamine (His; under lines). (a) Representative responses expressed as ΔR(CFP/YFP). (b) Histograms of maximum amplitudes (HeLa; n = 22, HeLa+H1; n = 32). White bars indicate cells showing no response.

In order to test the possibility that cell density affect calcium increase pattern by modulating inositol phospholipid metabolism, histamine-induced inositol phospholipid hydrolysis in HeLa cells were measured using a fluorescent resonance energy transfer (FRET) based imaging method [19]. Expression vectors containing fusion proteins of the pleckstrin homology domain, derived from rat PLCδ1, and cyan and yellow mutants of enhanced green fluorescent protein, were transfected into HeLa cells, and the interaction between these fusion proteins was determined by changes in FRET-based fluorescence (Fig 7). The histamine-induced fluorescence changes, were detected only in 4.5% and 0% of HeLa cells seeded at 1.5 x 10^4^ cells/cm^2^ and 0.5 x 10^4^ cells/cm^2^ respectively, whereas 90.6% of HeLa cells (1.5 x 10^4^ cells/cm^2^) expressing exogenous histamine H1 receptor showed robust fluorescence changes. ΔR(YFP/CFP) of cells expressing the exogenous receptor (0.301 ± 0.043, n = 32 cells) was significantly larger than the native cells (0.060 ± 0.003, n = 22 cells, p<0.01). Since histamine induced calcium increases in almost all native HeLa cells in the Fig 1 experiments, these results indicate that the endogenous histamine receptor in HeLa cells of any calcium increase patterns is capable of inducing limited hydrolysis of cellular inositol phospholipid.

### Effects of stimulus intensity

**Fig 8:**
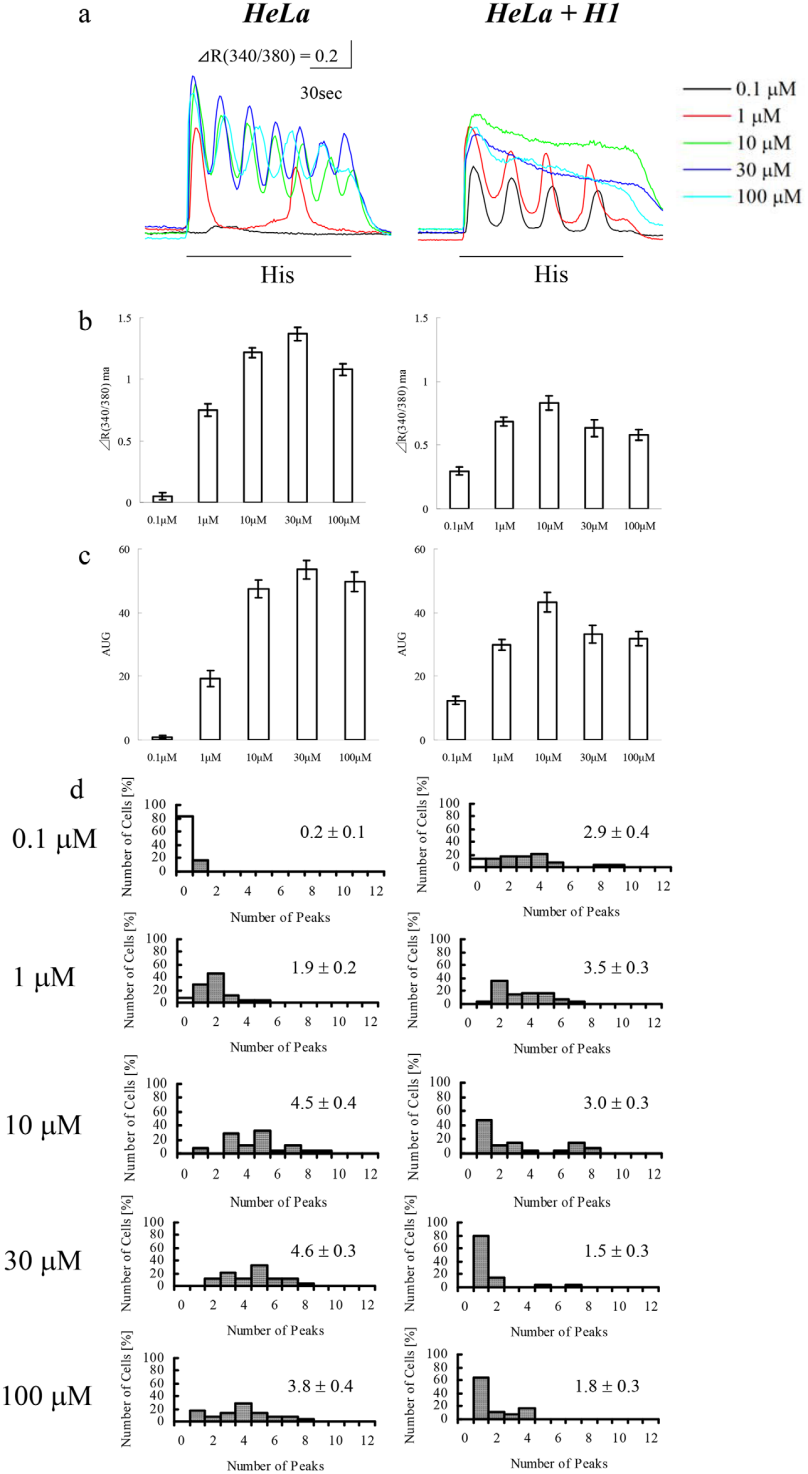
Effects of histamine concentration on calcium increase pattern. Calcium increases were induced in native HeLa cells seeded at 1.5 x 10^4^/cm^2^ (1.5 x 10^4^) or 0.5 x 10^4^/cm^2^ (0.5 x 10^4^), and HeLa cells (1.5 x 10^4^/cm^2^) expressing exogenous H1 receptor and GFP (HeLa + H1) by histamine at the indicated concentrations. (a) Representative calcium increase patterns in response to histamine stimulation (His; under lines). (b) Amplitudes of maximum calcium increase. (c) Integrated calcium increase (area under the graph, AUG). (d) Number of peaks in the calcium response. White bars indicate cells showing no response. Numerical values in each panel are average peak numbers (n = 28 cells each).

In order to examine the correlation between calcium increase pattern and the extent of inositol phospholipid hydrolysis, concentration dependency of histamine-induced calcium increases in HeLa cells seeded at 1.5 x 10^4^/cm^2^ with or without exogenous H1 receptor expression were compared (Fig 8). 0.1 μM histamine failed to induce calcium increase in 82% native HeLa cells, but only in 14% in HeLa cells expressing with exogenous H1 receptor and GFP as a transfection marker. In non-transfected HeLa cells, the portion of responding cells increased as histamine concentration increased. All parameters of calcium oscillations of native HeLa cells, including peak amplitude; integrated calcium increase, as indicated by the area under the graph; and the peak number of calcium oscillations saturated at 10 μM histamine, with no further increase observed up to 100 μM. In HeLa cells expressing exogenous H1 receptor, the calcium release pattern changed from oscillatory to sustained between 1–10 μM histamine, and peak amplitude and integrated calcium release were maximal at 10 μM. Above 30 μM histamine, calcium increase pattern became sustained, and peak amplitude and integrated calcium release decreased. These results indicate that exogenous H1 receptor, which robustly increases inositol phospholipid hydrolysis, enhances the sensitivity of HeLa cell to histamine and converts calcium increase pattern from oscillatory to sustained at high histamine concentrations. Since the sensitivity to histamine was not affected by seeding density and calcium increase pattern was conserved at histamine concentrations tested, in native HeLa cells, modulation of inositol phospholipid hydrolysis is unlikely the mechanism, by which cell density affects calcium increase pattern.

## Discussion

We have shown here that seeding cell density affected the calcium release patterns of HeLa and HEK293 cells. At 1.5 x 10^4^ cells/cm^2^, both of these cell lines showed calcium oscillation in response to ATP, whereas, at 0.5 x 10^4^ cells/cm^2^, their calcium release patterns were transient and sustained. Histamine-induced calcium release in HeLa cells was similarly affected by seeding cell density. An MEK inhibitor suppressed the calcium oscillation of HeLa cells seeded at 1.5 x 10^4^ cells/cm^2^. The calcium store content in HeLa cells was larger in cells seeded at 1.5 x 10^4^/cm^2^ than in cells seeded at 0.5 x 10^4^/cm^2^ or in cells seeded at 1.5 x 10^4^ cells/cm^2^ and cultured in the presence of the MEK inhibitor, indicating that enlargement of calcium stores correlated well with the tendency towards calcium oscillations. The influence of cell density, which affects calcium increase pattern, on inositol phospholipid hydrolysis was also investigated using a FRET-based imaging of inositol phospholipid breakdown. Histamine-induced inositol phospholipid hydrolysis was mostly below detectable levels in native HeLa cells, whereas, substantial in HeLa cells expressing exogenous H1 receptor. Exogenous H1 receptor also affected the calcium release pattern; i.e. non-transfected HeLa cells showed calcium oscillations even at high histamine concentrations, whereas HeLa cells expressing exogenous H1 receptor responded to 0.1 μM histamine, a concentration at which non-transfected HeLa cells failed to respond, and showed sustained calcium release at higher histamine concentrations. The sustained calcium release in HeLa cells expressing exogenous H1 receptor is most likely different from that in HeLa cells seeded at 0.5 x 10^4^/cm^2^, because the latter showed neither calcium oscillation, at lower histamine concentration, nor detectable IP_3_ production.

Although the increase in the ERK phosphorylation level in HeLa cells was correlated with calcium oscillation, the mechanism by which high cell density affects the ERK phosphorylation level is not yet known. It has been reported that HeLa cells utilize TGFα, which activates EGF receptors as an autocrine growth factor [23], however our pharmacological analyses have excluded the involvement of EGF-related growth factors, PDGF and bFGF in the up-regulation of calcium oscillation. We also failed to promote calcium oscillation in HeLa cells at low cell density using medium conditioned by cells at high cell density (data not shown), suggesting either that an autocrine factor is not utilized by HeLa cells or that it is easily degraded. The inhibitions of calcium oscillations by Genistein and PP2 indicate the involvement of Src-family tyrosine kinases, which mediate the MAP kinase activations by receptor tyrosine kinases (RTK), focal adhesion kinases (FAK), and cytokine receptors. We tested FAK inhibitors on the calcium oscillation, however HeLa cells did not survive during the long-term treatment as previously reported [24]. We assume that high cell density activates Src-family tyrosine kinases by altering intracellular redox state, which mediates RTK and FAK signalings [25]. Redox state is an activation mechanism of tyrosine phosphorylation-dependent signal transduction pathways [26], and it has been shown that intracellular redox state changes depending on cell density and growth [27]. The involvement of redox states in the regulation of calcium oscillation will be addressed by redox imaging and pharmacological modulation of redox states.

We found that MAP kinase activity correlated with stored calcium levels and altered calcium oscillation. The cellular mechanisms that regulate these calcium stores have yet to be elucidated. EGF induced growth of prostate cancer LNCaP cell has been associated with the expression of sarcoendoplasmic reticulum calcium ATPase 2b (SERCA2b) and the calcium pool content, measured by thapsigardine-induced calcium release [28]. Although we could not detect differences in SERCA2b expression between HeLa cells cultured at different cell densities, using RT-PCR (data not shown), we assume that expression of similar growth-related genes are responsible for the calcium oscillation in cell lines.

The present study provides a novel experimental model for studying cellular and molecular mechanisms underlying calcium oscillations. Larger calcium store content is assumed to lead to larger calcium release and higher calcium concentration close to IP_3_ receptor. Since the IP_3_ receptor is known to show a bell-shaped calcium dependency [29], larger calcium release is supposed to elicit dynamic behavior of the receptor. Calcium concentration in releasable stores has been reported to affect the IP_3_ receptor [30], so the calcium store contents presumably alters the kinetics of calcium release. These possible explanations for calcium oscillation will be examined in future studies, by comparing HeLa cells at different cell densities, and the parameters critical for calcium oscillation will be identified.

IP_3_ oscillations have been suggested to correlate with calcium oscillations in several model systems [14, 31], but it was not observed in the present study. Since there have been no studies comparing IP_3_ oscillation in cells positive and negative for calcium oscillation, it is not yet clear if IP_3_ oscillation is a cause or consequence of calcium oscillation. It is likely that only limited IP_3_ production is necessary for calcium oscillation, as proposed both in a theoretical study [32] and in an IP_3_ imaging study using a similar GFP-pleckstrin homology domain fusion protein [14]. At low stimulus intensity, a variety of cells show calcium oscillation [16, 33], similar to that observed in HeLa cells with or without exogenous H1 receptor. Due to the low level of histamine receptor expression in HeLa cells, limited IP_3_ production and calcium oscillation are thought to occur, even at high stimulus intensity. Since the calcium release pattern reflected both receptor expression level and stimulus intensity, the magnitude of receptor signaling was likely related to the quality (pattern) of calcium signaling.

The present study provides new basic information on calcium oscillation in HeLa cells, a model system used to study calcium dynamics. Our pharmacological and biochemical analysis indicates that basal MAP kinase activity, which is essential for cell growth at high cell density, enlarges calcium store and up-regulates calcium oscillation. The regulation of calcium oscillation by the cell density was observed in HeLa cells, and HEK293 cells, suggesting that this mechanism may be common to many non-excitable cells. Our findings may enable the establishment of conditions for the induction of reproducible calcium oscillation in many other cell types, thus accelerating progress in this field. Among the most important issues for understanding the mechanisms underlying calcium oscillation are the determination of the gene products involved in the regulation of calcium store content, and the ability to correlate calcium store content, which was quantified using ionomycin-induced calcium release, to calcium concentrations in the stores or the amount of available endoplasmic reticulum. In conclusion, our findings indicate that calcium store content and receptor expression level are useful parameters for understanding calcium dynamics in cells. Since these parameters are sensitive to the environment, they may cause heterogeneity in calcium release patterns.
